# Supplementary material for: The Role of Transient Crosslinks in the Chromatin Search Response to DNA Damage
Source: Int J Mol Sci. 2025 Dec 3;26(23):11697. doi: 10.3390/ijms262311697 (PMC12692392; doi:10.3390/ijms262311697)
Supplement: Supplementary file 1 [file ijms-26-11697-s001.zip › Supplementary Files and Captions.pdf]

**SUPPLEMENTARY MATERIALS:**

**Table S1:** *Relevant simulation time values.* In the mathematical model, we simulate chromosome movement for a total of 3700 seconds. We use data generated between 2400 to 3700 seconds after the start of the simulation, saved every 0.1 seconds. Other than sampling rate which is in units of seconds, the values included in this table correspond to "number of seconds after the start of the simulation".

| Data collection begins | Data collection ends | Sampling rate | Time of DSB induction | Time of localized crosslink inactivation |
|---|---|---|---|---|
| 2400s | 3700s | 0.1s | 2500s | 2500s |

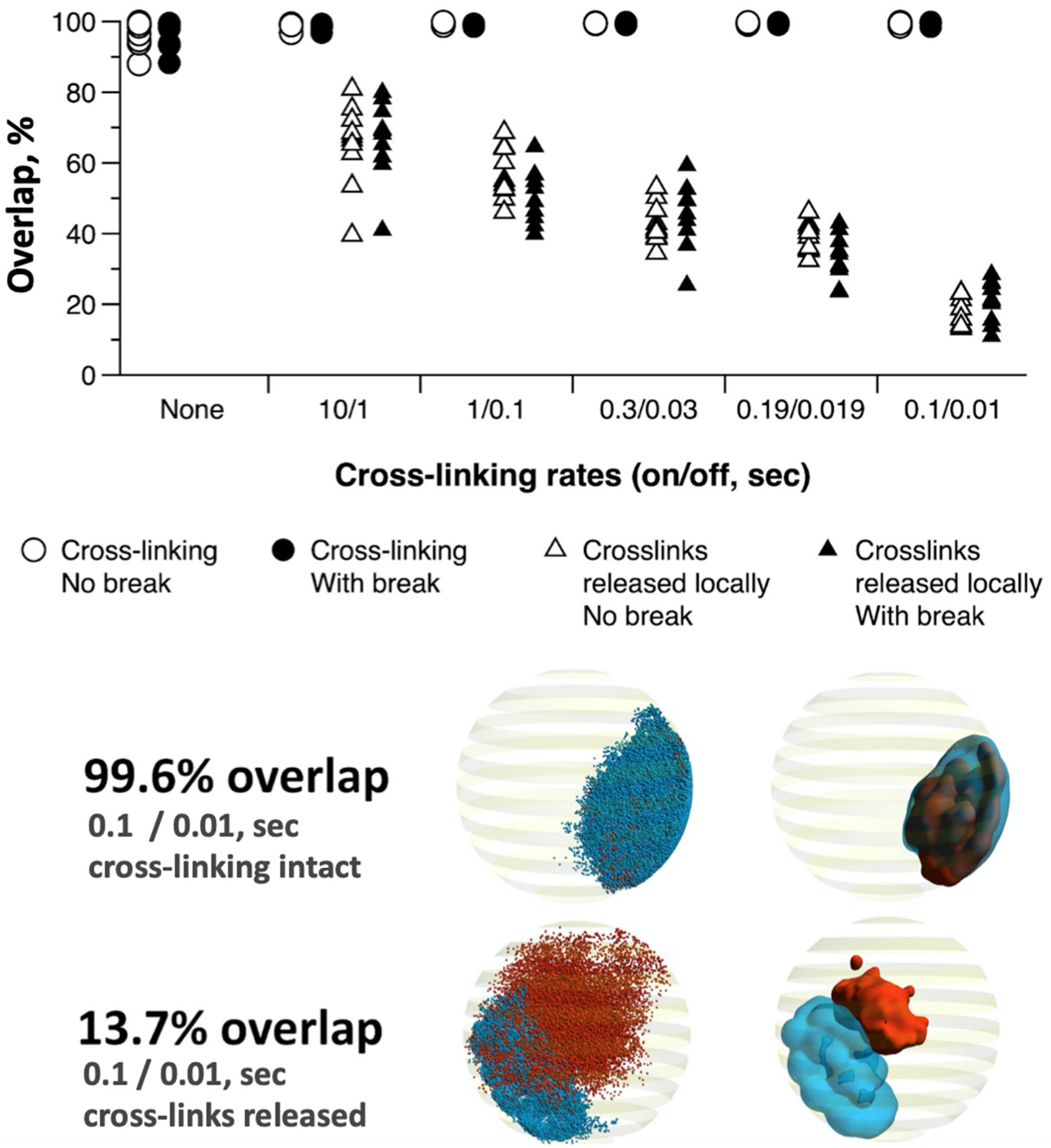


**Figure S1:** Quantification of the overlap between the damaged region with released cross-links (orange) and the remaining cross-linked region of the ROX region at different crosslinking regimes. Release of cross-links at t = 2500 s causes spatial separation of the damaged region from the rest of the ROX domain. Lowest overlap (i.e., highest degree of spatial separation) is observed at faster crosslinking regimes (0.19/0.019 and 0.1/0.01). Snapshots of high and low overlap are shown on the bottom as beads (left) and surfaces (right) corresponding to regions of high bead density.

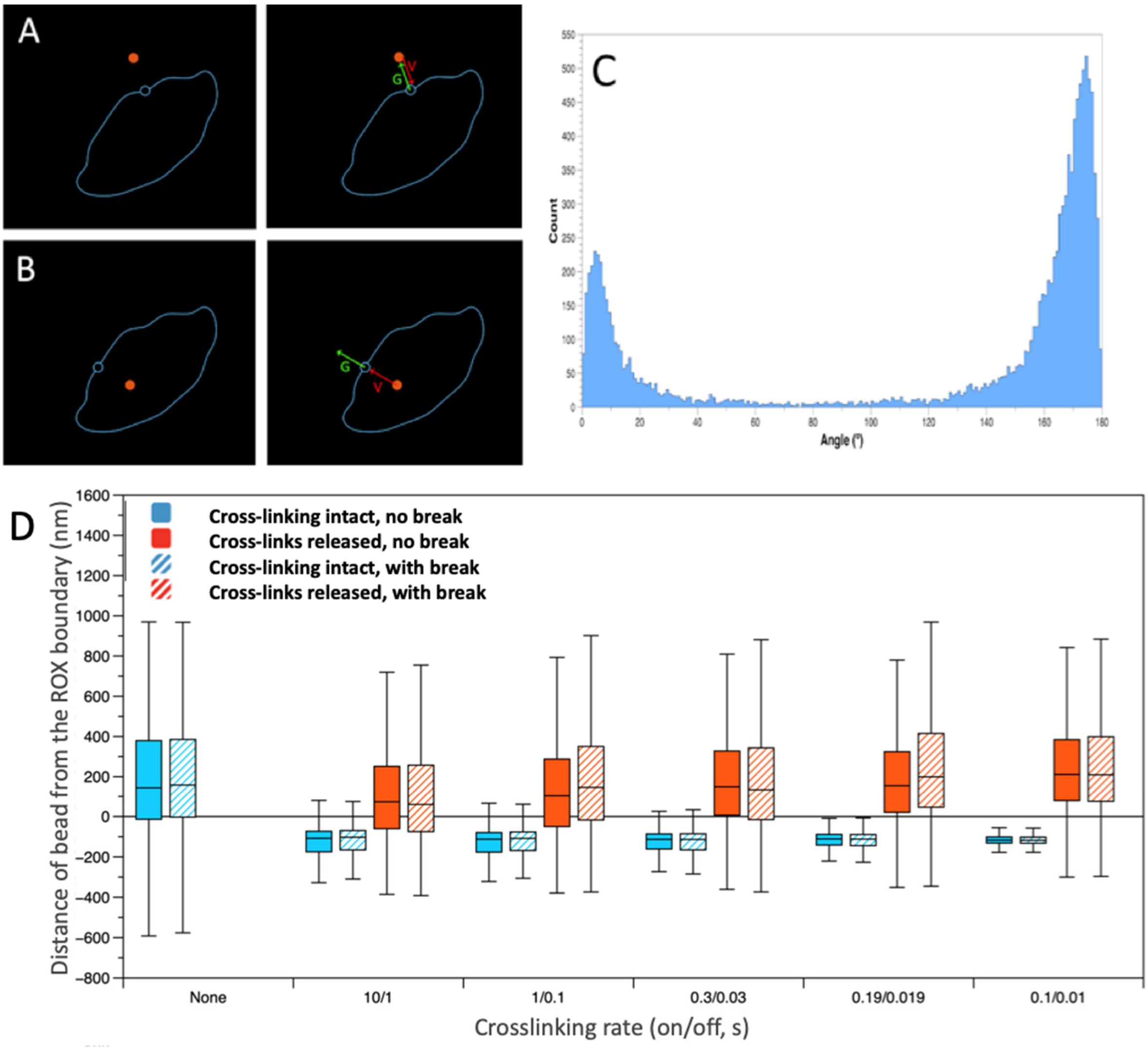


**Figure S2:** A different method for computing bead distance from the ROX boundary yields identical results. **(A-B):** The distance vector between the bead and the closest vertex on the 3D surface of the ROX domain (V) is compared with the gradient vector from the closest vertex (G). Because vector G points in the direction of decreasing point-spread function gradient, it will always be approximately perpendicular to the surface of the ROX domain. In panel A, vectors V and G point in opposite directions (i.e., the angle between them is close to 180 degrees), the bead is registered as being outside the ROX domain; in panel B, the angle between vectors V and G approaches 0, which indicates the bead is inside the ROX domain. **(C):** Distribution of the angles between vectors V and G across 10 simulations. The distribution is strongly bimodal, with the majority of angles concentrated around 0 and 180 degrees respectively, indicating this method is a viable approach for determining the presence of a bead within the ROX domain. **(D):** This method was applied to the full set of runs, with identical results to those shown in Figure 2.

**Video S1.** *Contact map of pairwise distances between ROX beads (on left) and ROX bead positions inside the nucleus (on right) over time corresponding to the ROX under the "fast" (0.1/0.01 s) crosslinking regime.* On the left, the x,y-axes in the contact map correspond to beads of the ROX (i.e., beads 61–421 on right arm of chromosome XII) from a single simulation. The gradient bar depicts distances in nanometers (also, see Figure 5). On the right, damage-induced depletion of cross-links at 2500 s in the region of ROX is depicted by red beads (beads 231–250). Blue beads are eligible for crosslinking; red beads lose the ability to crosslink; larger green beads (beads 240 and 241) lose the ability to crosslink and border the DSB site, were a DSB to occur. Each frame corresponds to 5 seconds of simulation. The ROX remains as an intact subcompartment throughout the simulation visualized by density of bead contacts on the map (on left) and clustering of blue beads via active crosslinking (on right).

**Video S2.** *Contact map of pairwise distances between ROX beads (on left) and ROX bead positions inside the nucleus (on right) over time corresponding to the ROX under the "intermediate" (0.19/0.019 s) crosslinking regime.* On the left, the x,y-axes in the contact map correspond to beads of the ROX (i.e., beads 61–421 on right arm of chromosome XII) from a single simulation. The gradient bar depicts distances in nanometers (also, see Figure 5). On the right, damage-induced depletion of cross-links at 2500 s in the region of ROX is depicted by red beads (beads 231–250). Blue beads are eligible for crosslinking; red beads lose the ability to crosslink; larger green beads (beads 240 and 241) lose the ability to crosslink and border the DSB site, were a DSB to occur. Each frame corresponds to 5 seconds of simulation.

**Video S3.** *Contact map of pairwise distances between ROX beads (on left) and ROX bead positions inside the nucleus (on right) over time corresponding to the ROX under the "slow" (10/1.0 s) crosslinking regime.* On the left, the x,y-axes in the contact map correspond to beads of the ROX (beads 61–421 on right arm of chromosome XII) from a single simulation. The gradient bar depicts distances in nanometers (also, see Figure 5). On the right, damage-induced depletion of cross-links at 2500 s in the region of ROX is depicted by red beads (beads 231–250). Blue beads are eligible for crosslinking; red beads lose the ability to crosslink; larger green beads (beads 240 and 241) lose the ability to crosslink and border the DSB site, were a DSB to occur. Each frame corresponds to 5 seconds of simulation.
